# Olive fly transcriptomics analysis implicates energy metabolism genes in spinosad resistance

**DOI:** 10.1186/1471-2164-15-714

**Published:** 2014-08-25

**Authors:** Efthimia Sagri, Martin Reczko, Maria-Eleni Gregoriou, Konstantina T Tsoumani, Nikolaos E Zygouridis, Klelia D Salpea, Frank G Zalom, Jiannis Ragoussis, Kostas D Mathiopoulos

**Affiliations:** Department of Biochemistry and Biotechnology, University of Thessaly, Ploutonos 26, Larissa, Greece; Institute of Molecular Biology and Genetics, Biomedical Sciences Research Centre “Alexander Fleming”, Athens, Greece; Department of Entomology, University of California, Davis, CA USA; Department of Human Genetics, McGill University, Montreal, QC Canada

**Keywords:** Insecticide tolerance, Spinosyns, Next generation sequencing, Expression analysis

## Abstract

**Background:**

The olive fly, *Bactrocera oleae*, is the most devastating pest of cultivated olives. Its control has been traditionally based on insecticides, mainly organophosphates and pyrethroids. In recent years, the naturalyte spinosad is used against the olive fly. As with other insecticides, spinosad is subject to selection pressures that have led to resistance development. Mutations in the α6 subunit of the nicotinic acetylcholine receptor (nAChR) have been implicated in spinosad resistance in several species (e.g., *Drosophila melanogaster*) but excluded in others (e.g., *Musca domestica*). Yet, additional mechanisms involving enhanced metabolism of detoxification enzymes (such as P450 monooxygenases or mixed function oxidases) have also been reported. In order to clarify the spinosad resistance mechanisms in the olive fly, we searched for mutations in the α6-subunit of the nAChR and for up-regulated genes in the entire transcriptome of spinosad resistant olive flies.

**Results:**

The olive fly α6-subunit of the nAChR was cloned from the laboratory sensitive strain and a spinosad selected resistant line. The differences reflected silent nucleotide substitutions or conserved amino acid changes. Additionally, whole transcriptome analysis was performed in the two strains in order to reveal any underlying resistance mechanisms. Comparison of over 13,000 genes showed that in spinosad resistant flies nine genes were significantly over-expressed, whereas ~40 were under-expressed. Further functional analyses of the nine over-expressed and eleven under-expressed loci were performed. Four of these loci (Yolk protein 2, ATP Synthase FO subunit 6, Low affinity cationic amino acid transporter 2 and Serine protease 6) showed consistently higher expression both in the spinosad resistant strain and in wild flies from a resistant California population. On the other side, two storage protein genes (HexL1 and Lsp1) and two heat-shock protein genes (Hsp70 and Hsp23) were unfailingly under-expressed in resistant flies.

**Conclusion:**

The observed nucleotide differences in the nAChR-α6 subunit between the sensitive and spinosad resistant olive fly strains did not advocate for the involvement of receptor mutations in spinosad resistance. Instead, the transcriptome comparison between the two strains indicated that several immune system loci as well as elevated energy requirements of the resistant flies might be necessary to lever the detoxification process.

**Electronic supplementary material:**

The online version of this article (doi:10.1186/1471-2164-15-714) contains supplementary material, which is available to authorized users.

## Background

The olive fruit fly, *Bactrocera oleae* (Rossi) (Diptera, Tephritidae) is the most important pest of cultivated olives. The female insect deposits its eggs in the olive fruit mesocarp where the developing larvae feed and grow. Furthermore, oviposition provides entry points for bacteria and fungi, increasing the consequences of damage. As a result olives either drop before maturity and become inedible or oil quality is affected [1]. More than 30% annual olive crop losses are attributed to the olive fly [2], which accounts to an economic impact of more than 800 million dollars [3].

During the last fifty years, the control of the fly has been traditionally based on chemical insecticides, mainly organophosphates (OPs) and, more recently, pyrethroids. Apart from the harmful effects of pesticides in the environment, insecticide exposure has led to the selection of resistant alleles in natural populations and the development of widespread insecticide resistance, mainly to organophosphates [4] but also to pyrethroids [5]. The mechanism of resistance to OPs has been extensively studied and has been attributed to target site mutations in the acetylcholinesterase (AChE). Two of these are point mutations that reside in the catalytic gorge of the enzyme [6] and a third one is a small deletion located in the carboxyl-terminal of the enzyme [7, 8].

Replacement of organophosphates with other environmentally friendlier products such as spinosad, has been a trend in recent years. Spinosad belongs to the naturalyte class [9] and has demonstrated particular efficiency against the Tephritid family of insects [10]. It is derived from the bacterium *Saccharopolyspora spinosa*, and is composed of a mixture of two macrocyclic lactones, spinosyn A (50-95%) and spinosyn D (5-50%) [9]. This insecticide acts by two main routes. Firstly, by activating the nicotinic acetylcholine receptor, but at a different site from that used by nicotine and imidacloprid [11], and secondarily through the GABA receptor, but at a distinct site from that used by abamectin [12, 13]. Spinosad may enter the organism by contact or through ingestion. The symptoms are limp paralysis, tremors and finally insect death [14].

Despite the relatively short history of spinosad in the marketplace, spinosad-associated resistance has been reported in many insects [15]. The first reports of spinosad resistance in the field were for the beet armyworm, *Spodoptera exigua* [16, 17]. Spinosad resistance has also been reported in several other species, such as the melon fly, *Bactrocera cucurbitae* [18], the Colorado potato beetle, *Leptinotarsa decemlineata* [19], the housefly, *Musca domestica* [20] and the tobacco budworm, *Heliothis virescens* [21]. The molecular mechanism of resistance to spinosad has not been fully clarified. There is evidence that resistance is a result of either enhanced metabolism of detoxification enzymes or a consequence of changes in a target site. The most noticeable target site of spinosad resistance is the nicotinic acetylcholine receptor (nAChR). In the case of *Drosophila melanogaster,* mutations in the α6 subunit of the nAChR (Dα6) confer high-fold resistance to spinosad, clearly implicating the Dα6 subunit in resistance [22, 23]. The α6 subunit of nAChR has been associated in spinosad resistance in other insects as well. For example, mis-spliced or truncated nAChR-α6 transcripts in the diamondback moth, *Plutella xylostella* [24, 25], truncated *Bdα6* transcripts of *Bactrocera dorsalis* [26], or a point mutation (*G275E*) in the transmembrane domain of the nAChR-α6 subunit in the western flower thrips, *Frankliniella occidentalis* [27], all confer high levels of resistance to spinosad. In contrast, spinosad resistance in *Musca domestica* does not seem to be related with the α6 subunit of nAChR. Instead, it correlates with a recessive factor on chromosome I [20], rather than the three nicotinic acetylcholine subunits (α5, α6, β3) that reside on the same chromosome [28].

In other cases, however, enhanced metabolism of detoxification enzymes have been implicated in spinosad resistance. For example, the microsomal-O-demethylase as well as monooxygenases were shown to be involved in resistance in *Spodoptera exigua* from China [29], an increase in cytochrome P450 monooxygenase was associated in cotton bollworm, *Helicoverpa armigera* [30], while enhanced activity of detoxifying mixed-function oxidases were connected with resistance in the Chilean populations of *Tuta absoluta* [31].

Until now, the most frequently used approach for isolating genes from an organism with few genetic and molecular tools was through PCR amplification with heterologous primers of the respective genes of closely related species. This approach, however, is greatly biased and excludes the possibility of identifying either genes that do not have homology in other organisms, or loci responsible for mechanisms that have not been studied in relative species. A transcriptomics approach, instead, may assess the differences of all expressed genes at the same time between sensitive and resistant individuals, without any preconceived ideas about specific genes, and thus reveal novel mechanisms that might be involved in resistance.

In the present study, we determined the sequence of the α6 subunit of nAChR of both a sensitive and a spinosad resistant olive fly strain, in order to explore possible presence of resistance mutations. In addition, we compared the entire transcriptomes of these two strains, in search of unknown loci that might be involved in spinosad resistance. Differential expression observed in several genes was validated both in laboratory colonies and field collected flies.

## Results

### Cloning of the *B. oleae*nAChR subunit α6 gene

A total of 2,367 bp of the *Bactrocera oleae* nAChR α6 subunit (Boα6) cDNA sequence was obtained from a susceptible laboratory (LAB) and a spinosad-selected (SPIN) strain. Initially, the *B. dorsalis*-based primers Bdα6F and Bdα6R amplified a partial ~1,800 bp coding fragment and subsequent 5′- and 3′-RACE reactions unraveled a 5′-UTR region of 300 bp upstream the start codon and a 3′-UTR of 600 bp that ended in a poly-A tail.

**Figure 1:**
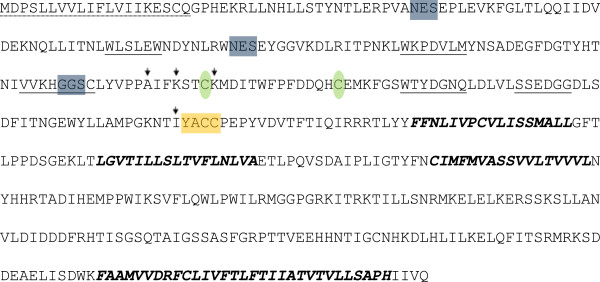
**Basic characteristics of the** ***Bactrocera oleae*** **nAChR α6 subunit.** N-terminal site is presented in dashed line and it is consisted of 20 amino acids. There are four transmembrane domains (TM1-4) (bold italic letters) and three glycosylation sites (blue boxes). The YxCC motif of alpha subunits is shown in orange box and the Cystein residues in green ovals. Six ligand binding loops are underlined. The three mutations are indicated by vertical arrows.

The beginning of the coding sequence was determined by the presence of a methionine residue at the expected place, as compared with known sequences from *Drosophila melanogaster* and *Bactrocera dorsalis*. Upstream of that residue there was no significant ORF. The next downstream Met residue was after 467 bp that would result in a substantially truncated product. An open reading frame of 1,467 bp encodes a putative 489 amino acid protein. The putative protein has 97% identity to the reciprocal *B. dorsalis* (AFN88980.1) protein. The Boα6 has all typical nAChR α subunit characteristics (Figure 1). The mature protein has a calculated molecular weight of 55.57 kDa and an isoelectric point of 4.49. It has all the characteristics of neurotransmitter-gated ion channels, with a signature of two cysteines separated by 13 amino acids [32] and four hydrophobic transmembrane domains (TM1-4) of conserved nAChR [33]. The Boα6 protein also possesses six loops and the alpha subunit character of YxCC motif [34].

Alignment of the two cDNA sequences obtained from the LAB and SPIN strains showed 13 point mutations (Additional file 1: Table S1). Ten of them were silent substitutions, while the remaining three led to homologous missense alterations: an Alanine (A) to Glycine (G) substitution at position 142 and two Lysine (K) to Arginine (R) substitutions at positions 145 and 149. The mutations are located in the N-terminal site and cause no changes on chain polarity or charge. In fact, the protein structure prediction server [35] indicated that the molecular structure of the receptor between the sensitive and the resistant strains remained unaffected. It is also known that nAChR α6 transcript undergoes RNA editing [36–38], although this process has not thus far been related to spinosad resistance. None of the 13 point mutations of Boα6 coincided with the recognized RNA editing sites of *Drosophila melanogaster* or *Bombyx mori*.

### Solid ABI sequencing and reads assembly

In order to explore possible mechanisms and pathways involved in spinosad resistance in *Bactrocera oleae,* the entire transcriptomes of the LAB and SPIN strains were compared. For transcriptome assembly, four libraries were sequenced and used. The sample names for the libraries are LAB, SPIN, MALE and FEMALE. Each library was sequenced with paired-end sequences, where each sequence pair consists of a 35 nt and a 50 nt fragment with a variable length insert between these fragments. Sequencing obtained a total of 122,623,894 read pairs. The reads of the libraries were pooled to construct a reference transcriptome assembly of 69,359 contigs using the SOAPdenovo assembler [39] (Table 1).

**Table 1:** **Sequencing and assembly statistics**

Sequencing and assembly summary		
Total number of paired-end reads		122,623,894
Total number of bases		10,423,030,990
LAB sample	number of paired-end reads	26,713,286
	number of bases	2,270,629,310
SPIN sample	number of paired-end reads	36,252,803
	number of bases	3,081,488,255
FEMALE sample	number of paired-end reads	36,962,061
	number of bases	3,141,775,185
MALE sample	number of paired-end reads	22,695,744
	number of bases	1,929,138,240
**Large contigs (≥500 bp)**		
Number of contigs		1,573
Number of bases		1,035,345
Average contig size		658
N50^*^		633
Largest contig size		2,301
**All contigs (≥100 bp)**		
Number of contigs		69,359
Number of bases		12,709,410

^*^Contig length, where all contigs of that length or longer sum up to at least half of the total of the lengths of all contigs.

### Sequence annotation

Annotation of the assembled sequences was obtained by aligning the 69,359 assembled *B. oleae* sequences against the NCBI non-redundant (Nr) protein database using blastx and collecting the annotations with the BLAST2GO tool [40]. Using an E-value threshold of ≤1e^-6^, 20207 (29.13%) of the contigs were aligned. The top 19 species in these alignments are diptera. Of the 69,359 contigs, 23,042 (33.22%) have almost exact hits in the *B. oleae* transcriptome of Pavlidi et al. [41] (E-value ≤1e^-6^).

### Only synonymous SNPs in detox genes

The presence of significant SNPs or truncations in known detoxification loci was assayed in the SPIN transcriptome. One hundred and fifty five genes involved in detoxification were analyzed. SNP calling was performed with the mpileup tool [42]. There are 9 SNPs in the sensitive strain (LAB) that are not in the resistant strain (SPIN), of which only 2 have more than 10 reads and were found to be synonymous. There are 19 SNPs in SPIN that are not in the LAB, of which only 2 have more than 10 reads and were found to be synonymous.

### Differentially expressed genes

The Cuffdiff [43] tool was used in order to reveal the differentially expressed genes between the spinosad resistant and the laboratory flies, a stringent cutoff (p value adjusted for multiple testing, called q value <0.05) was used. This resulted in 46 differentially expressed transcripts in the LAB vs. SPIN strain comparison.

**Figure 2:**
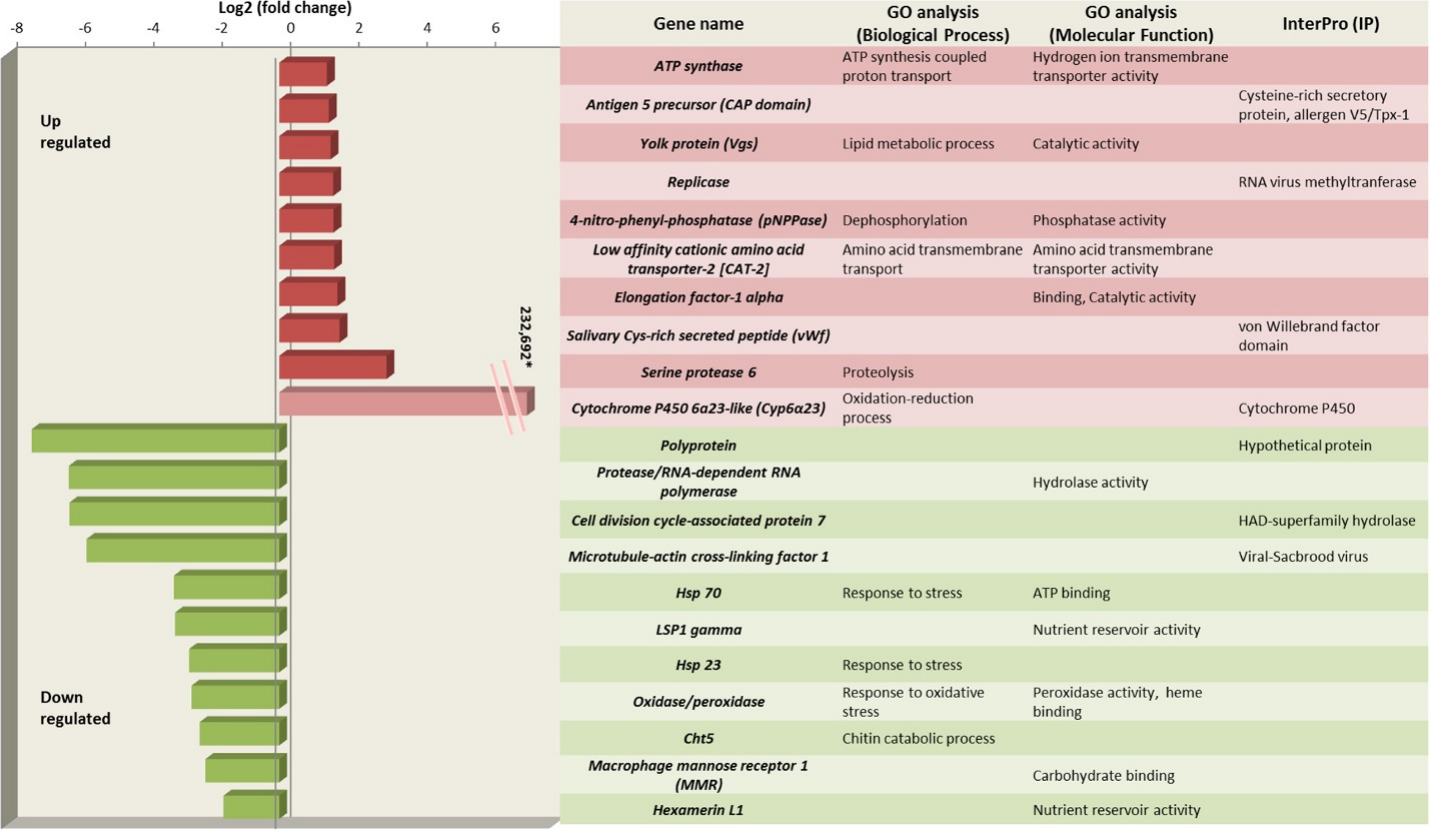
**Functional annotation of differentially expressed genes.** Gene expression levels of the differentially expressed genes (Log2, fold change), as resulted from the RNA-seq analysis, is shown at the left part of the Figure. Gene Ontology (GO) classification of the same genes for the ontologies: Biological Process (BP), Molecular Function (MF), and Interpro (IP) protein domains, are listed at the right part of the Figure. In crimson red are the up-regulated genes. The non-statistically significantly up-regulated *Cytochrome P450 6a23-like (Cyp6α23)* is shown in lighter color. In green are the down-regulated genes.

**Figure 3:**
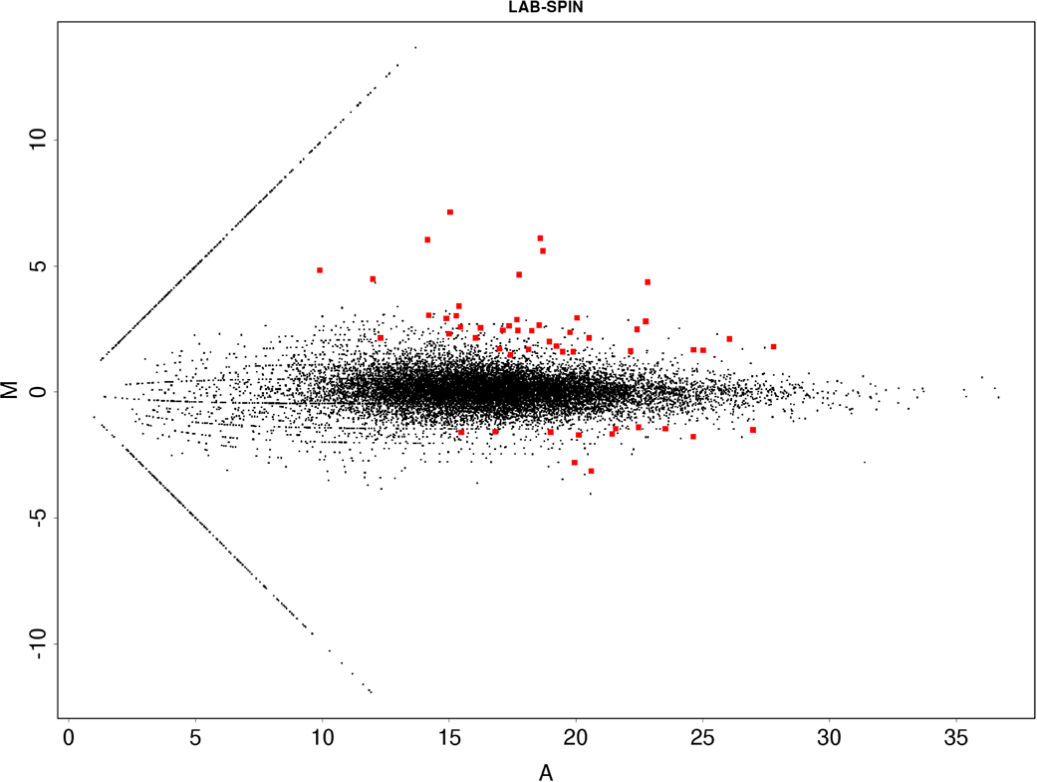
**M-A plot of the differential expression between sensitive and resistant flies.** The red color spots are the statistical significant differentially expressed genes with q-value < 0.05.

Twelve of these transcripts were up-regulated in SPIN resistant *B. oleae* flies than in sensitive (LAB) strain. More careful analysis revealed that three of these transcripts coincided with others and, therefore, nine distinct genes of the initial set of twelve were chosen for further functional analysis by quantitative real time PCR. These genes are listed in Figure 2 and Additional file 1: Table S2. Additionally, Cytochrome *P450 6α23-like* gene, a gene belonging in a group of known detoxification genes often involved in insecticide resistance, was considered. This gene was highly over-expressed, albeit not statistically significantly, falling below the stated criteria (p value = 0.000388, q value = 0.11).An M-A plot was also constructed for comparison of the genes for resistant vs sensitive strain with q value < 0.05. In Figure 3 the 12 up-regulated and 40 down-regulated genes in the resistant strain are depicted in red.

Finally, functional annotation was made for the assembled sequences of the significantly differential up- and down-regulated genes mentioned in Additional file 1: Table S2, based on gene ontology (GO) categorization obtained using BLAST2GO. The GO analysis performed for two main categories, molecular function and biological process, is shown in Figure 2.

### Functional analysis of genes that are putatively involved in spinosad resistance

In order to find the most suitable reference gene for the functional analyses of gene expression in the *B. oleae* head tissue, nine candidate genes were tested with NormFinder [44] and Bestkeeper [45] analysis. The nine genes were: *RPL19* (*ribosome protein L19*), *tbp* (*TATA-binding protein*), *ubx* (*ultrabithorax*), *GAPDH* (*glyceraldehyde 3-phosphate dehydrogenease*), *α-TUB* (*α-tubulin*), *β-TUB* (*β-tubulin*), *14-3-3zeta*, *RPE* (*RNA polymerase II*) and *actin3.* Normfinder analysis showed that the best housekeeping gene is the *14-3-3 zeta* with stability value 0.027 and the best combination of two genes is *tbp* and *14-3-3 zeta* with a stability value 0.031. From most stable (lowest stability value) to least stable (highest stability value) the candidate reference genes are ranged as: *14-3-3 zeta* < *ubx* < *tbp* < *β-TUB* < *GAPDH* < *actin3* < *RPE* < *RPL19* < *α-TUB*. These results were also confirmed by the Bestkeeper software since standard deviation and coefficient of variance values of *14-3-3 zeta* and *tbp* fell within the accepted range.

Functional analysis of all significantly over- or under-expressed aforementioned genetic loci was performed in conjunction with the best combination of the two housekeeping genes (*tbp* and *14-3-3 zeta*). Separately, the expression of all the target genes was calculated by normalization with *tbp* and *14-3-3 zeta*. The final expression value for each target gene was calculated as the geometric mean of its relative expression to the two housekeeping genes. Geometric mean values, range and standard error of expression are shown in Additional file 1: Table S3. More specifically.

#### Up-regulated genes

**Figure 4:**
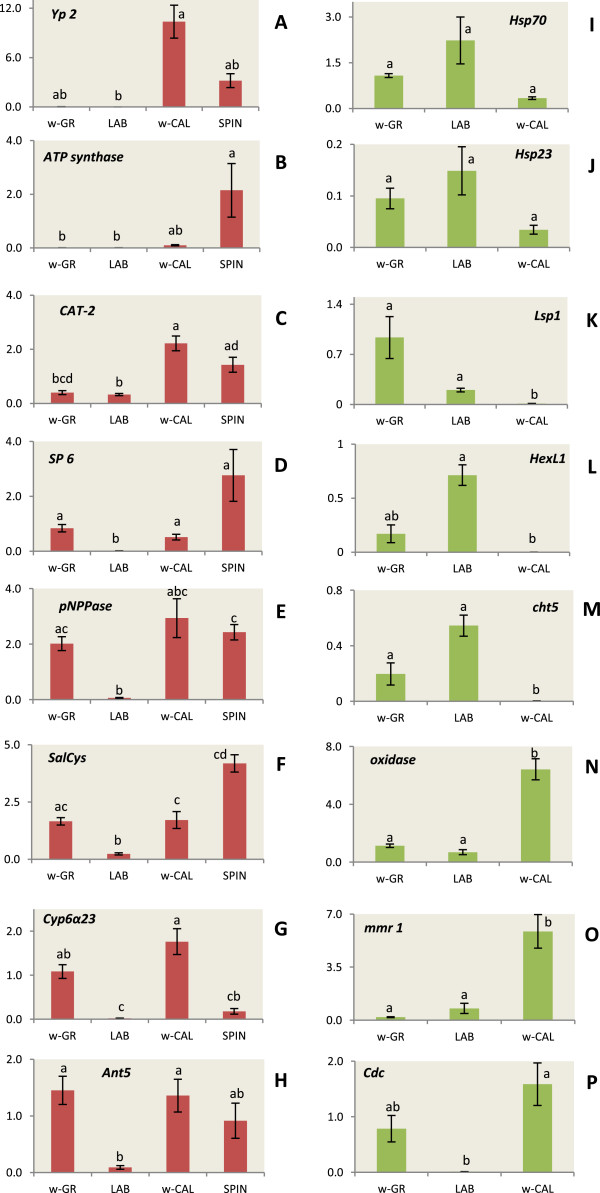
**Relative expression profiles of genes potentially associated with spinosad resistance.** The red color bars represent the up-regulated genes, *Yolk protein 2* (*Yp2*, Panel **A**), *ATP synthase F*_*O*_ *subunit 6* (*ATP synthase,* Panel **B**), *Low affinity cationic amino acid transporter 2* (*CAT-2,* Panel **C**), *Serine protease 6* (*SP6*, Panel **D**), *4-nitrophenylphophatase* (*pNPPase,* Panel **E**), *Salivary Cys-rich secreted peptide-*vWF (*SalCys*, Panel **F**), *Cytochrome P450 6a23-like* (*Cyp6α23,* Panel **G**) and *Antigen 5 precursor* (*Ant5*, Panel **H**), for the mean of three male and three female individual flies, after functional analysis by qRT-PCR. Only for the *Yolk protein* the evaluation was based on female expression, since males show zero expression values. The green color bars represent the down-regulated genes *Heat-shock protein 70* (*Hsp70,* Panel **I**), *Heat-shock protein 23* (*Hsp23,* Panel **J**), Larval serum protein 1 (*LSP1,* Panel **K**), *Hexamerin L1* (*HexL1*, Panel **L**), *Chitinase 5* (*Cht5,* Panel **M**), *Oxidase/peroxidase* (*oxidase*, Panel **N**), *Macrophage mannose receptor 1* (*mmr1*, Panel **O**), *Cell division cycle-associated protein 7* (*Cdc*, Panel **P**), for the mean of three male and three female individual flies, after functional analysis by qRT-PCR. The five RNA viral genes are not included. Standard error is also depicted in the bars. Small letters next to the error bars indicate significantly different mean values estimated by pairwise comparisons (either Tukey’s or Kruskal-Wallis tests). All comparisons were performed on Ln transformed data except for *macrophage mannose receptor 1*.

**Figure 5:**
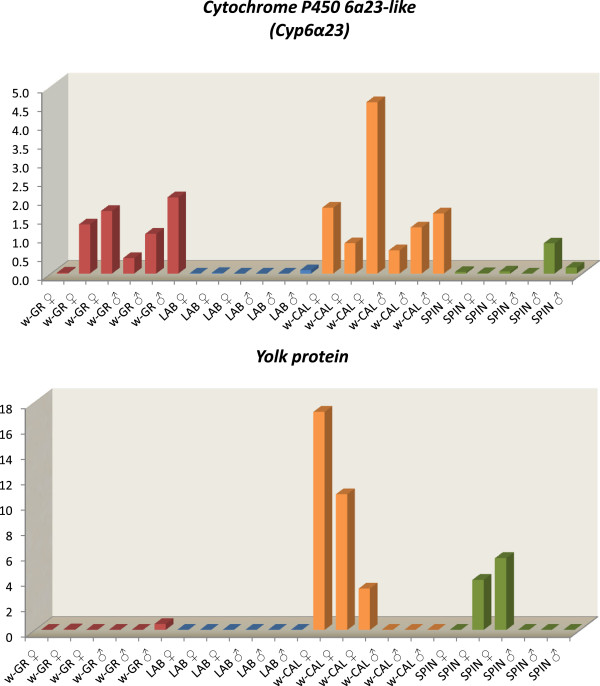
**Relative expression of** ***Cyp6α23*** **and** ***Yolk protein*** **in individual flies.** Relative expression of *Cyp6α23* **(Panel A)** and *Yolk protein 2* **(Panel B)** gene loci in the heads of individual olive flies of the w-GR (brown color bars), LAB (blue bars), w-CAL (orange bars) and SPIN (green bars) populations after functional analysis by qRT-PCR.

*Yolk protein 2* gene *(Yp2)* showed no relative expression in the sensitive flies (LAB, w-GR), while the expression in the resistant flies varied between 0.0075-5.656 and 3.265-17.178 fold for the SPIN and the w-CAL, respectively. As expected, the higher expression of spinosad resistance is observed only in female individuals, as *Yp2* is not expressed in males (Figures 4A and 5). Likewise, the relative expression of *ATP synthase F*_*O*_*subunit 6* in the sensitive flies of LAB and w-GR is approximately at the same range, nearby zero. The expression values in the two resistant groups (w-CAL, SPIN) were higher (Figure 4B), while a single male individual of the SPIN strain presented a remarkably high expression value (12.124-fold). Expression of *Low affinity cationic amino acid transporter 2* was 0.399-fold and 0.328-fold in w-GR and LAB, respectively, while expression in the resistant group was significantly elevated, 2.222-fold and 1.428-fold for w-CAL and SPIN (Figure 4C). *Serine Protease 6 (SP6)* was also significantly over-expressed in SPIN (2.763-fold) compared to the LAB (0.016-fold), while the expression of the wild flies was relatively low (0.838-fold for w-GR and 0.519-fold for w-CAL) (Figure 4D). The expression of *4-nitrophenylphosphatase (pNPPase)* was significantly higher in w-CAL as compared to LAB (2.937 vs 0.064), while that of w-GR was intermediate (2.016) but not significantly different from w-CAL (Figure 4E). The same pattern holds true for *Salivary Cys-rich secreted peptide* (vWF domain) and *antigen 5 precursor* (Figures 4F and 4H). Finally, for *cytochrome P450 6a23-like (Cyp6α23)* while the expression of SPIN was higher than LAB (0.179 vs 0.019) and w-CAL was higher than w-GR (1.762 vs 1.083), the differences were not statistically significant (Figure 4G).

#### Down-regulated genes

Functional analysis for the down-regulated genes was performed for LAB, w-GR and w-CAL populations since our SPIN colony was no longer available. Relative expression of both *Hsp* genes was not significantly different in the various groups of flies. *Hsp70* expression was 1.082-, 2.236- and 0.337-fold for w-GR, LAB and w-CAL, respectively, while *Hsp23* expression was 0.095-, 0.149- and 0.034-fold (Figure 4I and 4J). *Larval serum protein 1 (Lsp1),* on the other hand, was significantly under-expressed in w-CAL flies as compared to both w-GR and LAB (0.012 vs 0.937 and 0.203) (Figure 4K). Similarly, *Hexamerin L1* showed higher expression in the sensitive flies (LAB: 0.713, w-GR: 0.17), while for the resistant w-CAL the expression range was 0.001 (Figure 4L). Under-expression was even more pronounced in the *chitinase 5* locus of the resistant w-CAL (0.002) compared to both w-GR (0.197) and LAB (0.545) (Figure 4M). The expression pattern of *oxidase/peroxidase* did not confirm the RNAseq results, since expression of the resistant w-CAL was higher than that observed in the sensitive w-GR and LAB (6.148 vs 1.129 and 0.685) (Figure 4N). The same reverse pattern was observed for *Macrophage mannose receptor 1 (MMR)* (5.856 vs 0.196 and 0.776) and *cell division cycle associated protein7 (Cdc)* (1.585 vs 0.784 and 0.0102) (Figures 4O and P).

## Discussion

Spinosad is a relatively new and very promising insecticide used against a variety of insect pests. As is the case with any other chemical, resistance has already developed in several natural and greenhouse populations of insects. In several cases of resistance, mutations in the α6 subunit of the nAChR were shown to be responsible, while in others this locus was shown to not be involved. Yet, general detoxifying systems have also been implicated. In order to understand the mechanism of spinosad resistance in the olive fly, we both looked for mutations in the *Boα6* nAChR as well as searched the entire transcriptome for potential new loci involved in resistance.

Firstly, the *Boα6* cDNA from the olive fly *Bactrocera oleae* was identified and characterized. The deduced amino acid sequence presented very high similarity with α6 subunits of other diptera and retained typical subunit characteristics with the nAChR homologs. Comparison of the Boα6 between the laboratory sensitive (LAB) and spinosad-resistant (SPIN) strains yielded three homologous amino acid substitutions. This fact most likely excludes the involvement of Boα6 nAChR in resistance, at least under the conditions of the experiment. Indeed, it should be pointed out that all published reports that implicate α6 nAChR subunit in spinosad resistance, the resistance level of the organism is considerably high: ~1200-fold in *D. melanogaster* [22], >2000-fold in *B. dorsalis* [26], >350,000-fold in *F. occidentalis* [27], 1070-fold in *H. virescens* [21], 18,600-fold in *P. xylostella* [24, 25]. On the contrary, lower levels of resistance are associated with mechanisms that do not involve target site resistance but, rather, more generalized detoxification systems. This is the case, for example, of *M. domestica* (~150-fold; [20]), *H. armigera* (20-fold; [30]), *S. exigua* (~350-fold; [29]), *T. absoluta* (1.8 to 4.6-fold; [31]) and *B. oleae* (~35-fold; this study).

Secondly, in our efforts to shed light on other possible mechanisms of resistance, we compared the entire transcriptomes of a laboratory sensitive (LAB) and a spinosad resistant (SPIN) strain through RNAseq. During the course of our study, Pavlidi and co-workers produced a basic transcriptome dataset for *B. oleae* using 454 pyrosequencing [41]. Due to the different sequencing technology used in the present study, our reference transcriptome has fewer long contigs but a significantly higher number of total contigs and contigs with alignments (Table 1), which is more relevant for the purpose of detecting differentially expressed genes. Our comparative LAB vs SPIN analysis yielded several over- and under-expressed loci that are discussed below. Two caveats should be added at this point. Firstly, since LAB and SPIN transcriptomes were sequenced only once, we ought to validate the observed differences through qRT-PCR in multiple samples. In order to ascertain that the observed differences reflected differences that would hold true not only under laboratory but also under natural conditions, we decided to extend our validation experiments in natural spinosad-sensitive and spinosad-resistant populations. As such, wild flies were collected from a presumably untreated orchard in the surroundings of the city of Volos (Greece) (w-GR), where there is no documented use of spinosad and from a site in Sonoma County in California (w-CAL) with the highest documented naturally observed spinosad-resistance ratio (see Methods). However, resistance bioassays showed that while the resistance ratio (RR) of the SPIN strain was ~35, w-CAL and w-GR had RRs 12.96 and 3.14, respectively. Therefore, w-GR cannot be considered as a source of truly spinosad sensitive flies. Indeed, the expression of various genes was shown to be intermediate between LAB and SPIN values. Secondly, the presence of different resistance mechanisms in the laboratory or naturally selected flies cannot be excluded. While this is plausible, we doubt it for three main reasons: One, the genetic background of the SPIN strain and the w-CAL is similar since the SPIN strain was enriched by w-CAL; two, the selective force used in the laboratory was the same as the one used in California (i.e., spinosad); and, three, the difference between w-CAL RR and SPIN RR (~13 vs ~35) is not that large to indicate the presence of a different mechanism. As stated earlier, usually high RR levels are associated with target-site resistance while lower RR levels are associated with more generalized detox mechanisms. Be that as it may, and even if more resistance mechanisms are at play, our analysis should point towards their underlying common ground. And since spinosad selection is common between SPIN and w-CAL flies, any transcriptome differences with LAB (and partly w-GR) should indicate events involved in spinosad resistance.

**Figure 6:**
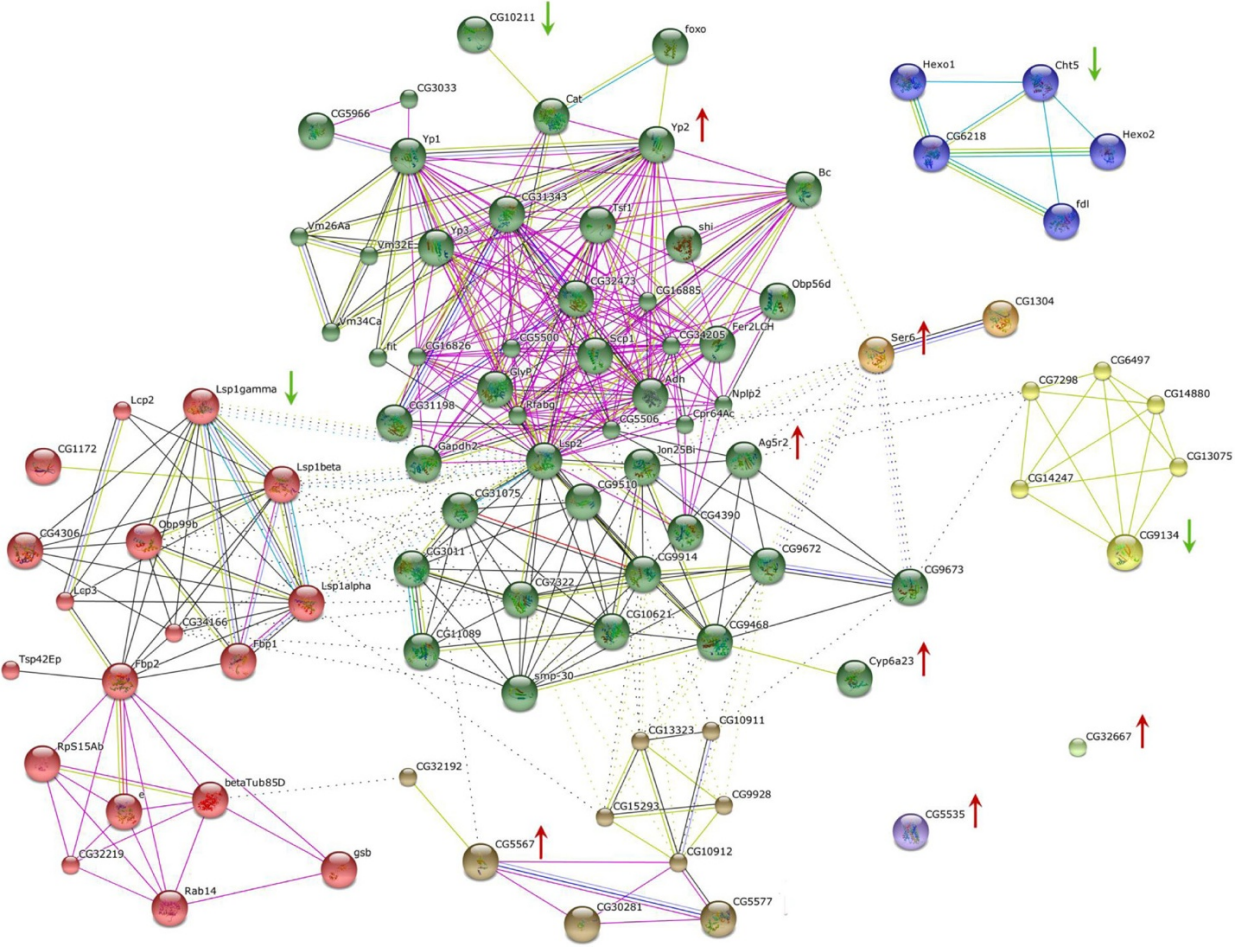
**STRING analysis.** The network displays the predictions of protein interaction and association with experimentally determined interactions plus those from the literature of the selected gene list of up-regulated as well as down-regulated gene-products. The input gene list included the following genes: *CAT-2* (CG5535), *Serine protease 6* (CG2071), *Yolk protein 2* (*Yp2*, CG2979), *pNPPase* (CG5567), *Lsp1* (CG6821), *oxidase* (CG10211), *MMR* (CG9134), *Cytochrome P450 6a23-like* (*Cyp6a23*, CG10242), vWF domain (CG32667) and *chitinase* (CG9307). Network was enlarged based on Drosophila protein interactions. The *ATP synthase gene* (CG8189) and the *Hsps* were withdrawn from the list, because the resulting network was very dense and uninterpretable. Interestingly, the gene *Ag5r2* (*Antigen 5*) even if it was also absent from the input list, it appears to be correlated with the other genes, supporting our hypothesis of interacting pathways. STRING Version 9.O was used for this analysis. Different colored edges indicate the types of evidence used in predicting the associations^1^. Up-regulated genes are indicated by red arrows, whereas down-regulated ones by green arrows. ^1^A red line indicates the presence of fusion evidence; a green line - neighborhood evidence; a blue line - coocurrence evidence; a purple line - experimental evidence; a yellow line - textmining evidence; a light blue line - database evidence; a black line - coexpression evidence.

Potential interactions between the up- and down-regulated genes were examined through STRING (Search Tool for the Retrieval of Interacting Genes). STRING makes available precomputed results in predicted functional linkages among proteins by comparative genomics and text-mining [46]. STRING analysis using the MCL clustering algorithm yielded several links between the examined differentially expressed genes (Figure 6). The generated network supports the hypothesis of non-randomness in the results and rather reflects a regulation feature by both activator genes (the up-regulated expression) and maybe also repressor genes (down-regulated expression). However, for the characterization of the transcriptional regulatory network and the understanding of these genes dynamic association and their possible involvement in insecticide resistance, we should first consider their function and their well-documented role.

### Increased energy and metabolism demands

ATP synthase is an important enzyme that provides energy for the cell to use through the synthesis of ATP. Located within the mitochondria, ATP synthase consists of 2 regions: the F_O_ portion is embedded in the mitochondrial membrane and functions as a proton pore; and the F_1_ portion is inside the matrix of the mitochondria and is associated with the ATP synthase activity. Through differential proteome analysis and enzyme activity assays, increased expression of ATP synthase was found in the midgut of pyrethroid resistant populations of *Helicoverpa armigera* [47]. Since more energy related proteins (such as vacuolar-type ATPase A/B and arginine kinase) were up-regulated, the authors suggested that increased energy metabolism may be a general prerequisite for compensating the costs of energy-consuming detoxification processes. As a matter of fact, inhibitors of mitochondrial ATP synthase, such as Diafenthiuron, are known insecticides for aphids, whiteflies and hoppers [48]. Significantly elevated levels of *ATP synthase F*_*O*_*subunit 6* were observed in SPIN flies as compared with LAB and w-GR, while w-CAL levels were intermediate (Figure 4B). This constitutes an indication of elevated energy requirements of the resistant flies so as to lever the detoxification process.

The Low affinity cationic amino acid transporter-2 (CAT-2) belongs to a large group of solute carrier proteins, a group of over 300 membrane transport proteins organized into 52 families [49]. These transporters utilize the energy of ATP hydrolysis to transport various substrates across cellular membranes. Several functions are controlled such as protein synthesis, hormone metabolism, catalytic functions, nerve transmission, regulation of cell growth, production of metabolic energy, synthesis of purines and pyrimidines, nitrogen metabolism and biosynthesis of urea. In addition, in the mammalian cells, the uptake of amino acids is mediated by energy-dependent and passive transporters with overlapping substrate specificities. Most energy-dependent transporters are grouped either to the co-transport of Na^+^ or Cl^-^ or to the counter-transport of K^+^. As reported for system y^+^, the CAT proteins catalyze the Na^+^-independent uptake of arginine, lysine and ornithine and the Na^+^-dependent uptake of some neutral amino acids [50]. Both SPIN and w-CAL olive flies showed significantly higher *CAT-2* levels compared to LAB and w-GR (Figure 4C). While there are no data in the literature suggesting that *CAT-2* may be involved in transport or extrusion of spinosad from the cell, we think that the up-regulation of this locus is related with the up-regulation of *ATP synthase* and reflects the increased energy and metabolic needs of the resistant flies.

### Egg and larval development proteins

Vitellogenins (Vgs) are precursors of the major egg storage protein, vitellin, in many oviparous animals. In higher Diptera, Vgs are called Yolk proteins (Yps) and are produced by both the fat body and the ovary in the majority of the species. Three main factors regulate vitellogenesis in *D. melanogaster*: a brain factor, an ovarian factor that stimulates fat bodies Yp synthesis (further recognized as ecdysone) and a thoracic factor (Juvenile Hormone, JH) involved in the Yp uptake by ovaries. JH regulates the Yp synthesis and uptake, while ecdysone is involved only in Yp synthesis [51, 52]. In Culex mosquitoes the head factor is released 4–8 minutes after the beginning of feeding [53]. The vitellogenic phase is initiated after feeding on non-autogenous species or after the adult emergence of autogenous species, when the *corpora cardiaca* stimulating factor (CCSF) is released from the ovary [54, 55]. Insect Vgs are synthesized in the fat body in a process that involves substantial structural modifications (e.g., glycosylation, lipidation, phosphorylation, de-phosphorylation, proteolytic cleavage, etc.) of the nascent protein prior to its secretion and transport to the ovaries (for a review see [56, 57]). *4-nitro-phenyl-phosphatase (pNPPase)* catalyzes the hydrolysis of nitrophenyl phosphates to nitrophenols. At acid pH it is probably acid phosphatase; at alkaline pH it is probably alkaline phosphatase. In the kissing bug *Rhodnius prolixus*, acid phosphatase activation follows oocyte fertilization and *pNPPase* seems to be involved in vitellin dephosphorylation [58]. Taken together, *pNPPase* should follow elevated levels in *Yp* expression since it is involved in its modification during transport to the ovaries (Figure 4E). In the case of spinosad resistant flies, the elevated levels of *Yp2* and, to a lesser extent of *pNPPase*, observed in the resistant flies is most likely related to events taking place in the fat body surrounding the heads of the insects rather than their brain and probably associated with feeding rather than processes associated to egg development. Furthermore, it has been reported that there is a physiological link between vitellogenin activity, oxidative damage and mortality, suggesting an antioxidant role of vitellogenin. RNAi experiments in bees demonstrated that vitellogenin expression was linked to the bees’ level of resistance to oxidative stress [59]. In the same study, excess mortality of *vg*^-^ bees was shown to be linked to cellular damage that included a severe oxidative insult to the fat body, after exposure to paraquat. Τhis elevated expression of *Yp2* gene in spinosad-resistant flies is somewhat analogous to the observance of persistent production of a vitellogenin-like protein in insecticide-resistant mosquitofish *Gambusia affinis*. Normally, in the mosquitofish Vg is produced during reproductive season. However, insecticide-resistant mosquitofish produce a vitellogenin-like protein year around [60]. The authors suggest that xenobiotics may induce the formation of a vitellogenin-like protein in order to bind and transport insecticides. Finally, we questioned whether the observed up-regulation is female-specific only. In fact, as expected, functional analysis in three female and three male flies of SPIN, w-CAL, LAB and w-GR showed elevated *Yp* expression in female SPIN and w-CAL heads only (Figure 5). Interestingly, the within population variability was very high. While values for w-GR and LAB were close to zero (0.0016-0.0548 and 0.00036-0.00079, respectively), values for w-CAL ranged from 3.265 to 17.178 and for SPIN 0.0075 to 5.656. Considering that all SPIN flies fed on constant spinosad diet, the low *Yp* expression observed in a SPIN female (0.0075) suggests that high *Yp2* expression may be protective but not necessary for spinosad resistance in female flies.

By contrast, two storage proteins, *hexamerin larval protein 1 (HexL1)* and *larval serum protein 1 (Lsp1)*, showed a tendency of down-regulation. In holometabolous insects, which go through distinct stages, essential nutrients obtained in one stage but required in another are sequestered in storage proteins and carried across stages until they are utilized. In that sense, if an insect does not feed or restricts its diet during a specialized stage, its activities should be supported by nutrient intake during a previous feeding stage. Egg development in mosquitoes, for example, heavily depends on a protein-rich blood feed. Nectar feeders, on the other hand, should obtain most protein destined for eggs during the larval stage and stored until synthesis of yolk proteins. This storage takes place through various structurally similar hexamerins (for a review, see [61–63]). Storage proteins are not only produced during larval development. Adult females of the diamondback moth, *Plutella xylostella*, synthesize hexamerins within hours post eclosion to resequester amino acids that have been utilized until then [64]. Hexamerins are also implicated in JH regulation. In termites, hexamerins are involved in nutrient storage and nutritional signaling and are also known to bind JH [65]. It is thought that by binding to it hexamerins sequester JH, thus preventing it from eliciting downstream effects on developmental gene expression [66]. Indeed, RNAi-based hexamerin silencing affected 15 out of 17 morphogenesis-associated genes that are members of a JH-responsive genomic network [67].

So, why are storage protein transcripts down-regulated in spinosad resistant flies? It is plausible that the resistant w-CAL flies (and to a lesser extent the less resistant w-GR flies) have developed the ability to store sufficient amounts of the necessary amino acids for their adult lives during their larval stages and to not require additional replenishments during adulthood. Such nutrient availability may be necessary for overcoming the elevated demands in energy and metabolism in the ‘toxic’ environment of the resistant flies. Instead, under ‘normal’ conditions, when the flies have the luxury of acquiring and store amino acids later in their adult lives, they can activate their storage proteins after a meal. In order to prove this claim, however, further detailed experiments should be performed to assess the expression of storage and related genes during the larval, pupal and adult stages, under different nutritional conditions.

### Immunity, detoxification and stress related loci

Six genes that fall in this category have raised our interest.

*Serine protease 6 (SP6).* While the role of other detoxification enzymes in insecticide resistance is well understood, the involvement of proteases/serine proteases is not. Proteases are involved in protein digestion outside the cells and also in the expression and regulation of cellular proteins [68]. Cellular proteases function to create biologically active molecules or destroy biologically active proteins and peptides [69, 70]. Additionally, the signalling transduction system/pathways that are controlled by G protein coupled receptors (GPCRs), protein kinase/phosphatases and proteases are involved in the regulation of *P450s* genes [71]. Very interestingly, elevated levels of all cytoplasmic and lysosomal proteases were detected in spinosad-resistant *M. domestica* flies 48 hours after exposure to spinosad at LD_50_ dose level [72], indicating involvement of proteases in the development of spinosad resistance to the housefly. Two serine protease genes (*trypsin* and *chymotrypsin*) were also shown to have threefold higher expression in deltamethrin-resistant *Culex pipiens pallens* mosquitoes [73]. These two enzymes were further shown to hydrolyze deltamethrin [74]. Moreover, up-regulation of serine proteases was also documented in permethrin resistant *Culex quinquefasciatus* mosquitoes [75]. Finally, in the mosquito *Aedes aegypti*, *serine proteases* are also expressed in the salivary glands and thought to have a defense role against bacterial growth ingested with saliva during sugar meals [76, 77]. In the olive fly, the level of *serine protease 6* in the resistant SPIN and w-CAL flies strain is significantly elevated compared to LAB (Figure 4D), while w-GR has also considerable expression. Apparently, *serine proteases* are required not only for the digestion of more complex nutrients of the wild flies, compared to the standardized laboratory diet, but may also participate in the defense against bacterial pathogens during feeding.

An oxidase/peroxidase family protein was found down-regulated in the transcriptome of the SPIN strain. However, further comparisons between LAB, w-GR and w-CAL reversed the trend and showed higher level of expression in w-CAL flies. While such proteins present protein-protein binding properties and are known to be involved in defense mechanisms (such as intracellular phagocytosis of apoptotic cells or foreign material) [78], the gene was not further evaluated.

A *macrophage mannose receptor (MMR)* was also found to be down-regulated in the SPIN strain. The MMR is a C-type lectin receptor, a family of surface carbohydrate-binding receptors that require calcium for binding. In humans they are known to recognize microbial carbohydrate moieties, also sense products from dying cells and transduce inflammatory signals that modulate the immune system [79]. In crustaceans, on the other hand, they are thought to be involved in the regulation of the exoskeleton calcification [80]. Its expression displayed molt cycle-related differential profile. In the same study, members of the serine protease superfamily also varied their expression during different molting stages. In insects, secretory C-type lectins are thought to play roles in cellular interactions during development [81]. In addition, they are considered important in the immune system, including the detection and neutralization of pathogenic and non-self materials in several insect species [82]. In the mosquito *Aedes aegypti* and the flesh fly *Sarcophaga peregrina*, C-type lectins are expressed in the salivary gland and are considered to control bacterial pathogens from ingested meals [76, 77, 83, 84]. In the olive fly transcriptome a *macrophage mannose receptor* was found to be down-regulated in the SPIN strain but the trend was reversed in the functional analysis of the LAB, w-GR and w-CAL strains and, therefore, cannot be evaluated before further analyses are performed.

A von Willebrand factor domain within a Salivary cys-rich peptide was also up-regulated. The majority of vWF-containing proteins are extracellular. The oldest ones in eukaryotes, however, are parts of intracellular proteins involved in transcription, DNA repair, ribosomal and membrane transport and the proteasome. vWF tends to bind to other proteins and thus it appears to be involved in multiprotein complexes. In insects, huge vWF-containing proteins, such as hemolectin in *D. melanogaster* and hemocytin in *B. mori*, are thought to function in the hemolymph coagulation or hemocyte aggregation processes, such as nodule formation [85, 86]. Such processes are fundamental responses of insect innate immunity in order to clear microorganisms from the hemocoel. A similar role might be envisaged in SPIN flies of *B. oleae*. This up-regulation is concordant with the up-regulation of the previously described defense loci. Functional analyses on LAB, w-GR and w-CAL flies confirmed a significant under-expression in the LAB strain (Figure 4F).

#### Cytochrome P450 6a23-like *(Cyp6α23)*

This gene belongs to a superfamily of monooxygenases that catalyze the oxidation of organic substances. They are involved in drug metabolism and bioactivation of about 75% of all the different metabolic reactions [87]. *P450s* have been implicated in insecticide resistance against various substances (for reviews see [88–90]). Their role in spinosad detoxification has been hypothesized at least in *Helicoverpa armigera* [91], *Musca domestica* [92] and *Bombus huntii* [93], whereas it has been disputed in *Drosophila melanogaster* [94]. *Cyp6α23* was highly over-expressed in the RNAseq of the olive fly SPIN strain (232,692-fold), albeit not statistically significantly, falling below the stated criteria (p value = 0.0003877, q value = 0.109514). Functional analysis in three female and three male flies of SPIN, LAB, w-CAL and w-GR showed, on average, elevated levels of expression in SPIN and w-CAL compared to LAB (Figure 5). However, w-GR had intermediate levels of expression. Two things should be mentioned at this point. Firstly, the large variability of *Cyp6α23* levels. In some SPIN individuals the *Cyp6α23* level was lower than that of some LAB individuals. However, since the RNA for the RNAseq was obtained from a pool of 40 female and 40 male flies, the RNAseq result should reflect the average expression in the population. In addition, *P450s* expression levels vary throughout the life cycle of the insect [93] and the observed variability in *Cyp6α23* expression in olive fly individuals may reflect the asynchrony of their life stage. Secondly, w-GR flies had, on average, intermediate levels of *Cyp6α23* expression. As mentioned in the Methods section, even though these flies were obtained from a presumably untreated orchard in Greece, their resistance ratio was three times higher than that of the LAB flies and, therefore, w-GR cannot be considered as a source of truly spinosad sensitive flies.

#### Heat shock proteins

Two heat shock proteins, Hsp70 and Hsp23, were found to be down-regulated in the SPIN transcriptome, a fact that was not confirmed after functional analyses. Hsp70 proteins are very conserved and ubiquitously expressed in virtually all living organisms, being very important in folding and unfolding of proteins, detoxification of pesticides and heavy metals. Hsp23 belongs to a lens alpha crystalline-related superfamily, also found in the salivary gland cells of *D. melanogaster* [95]. In all reported cases of stress and detoxification where Hsp were involved, their transcripts were strongly up-regulated. In order to clarify their role in spinosad resistance in the olive fly, further experiments should be performed.

#### Antigen 5 precursor (Ant5)

This gene product shows similarity to Drosophila’s *Antigen 5-related 2 gene (Agr2). Agr2* proteins belong to the CAP family of proteins, which include the mammalian Cysteine-rich secretory proteins, wasp venom Antigen 5 proteins, and plant group 1 Pathogenesis-related proteins. The gene product of the *Drosophila melanogaster* ortholog *Agr2* is suggested to function either as a novel type of protease inhibitor or as an antimicrobial protein [96]. In our study, *Ant5* was over-expressed in the SPIN transcriptome. However, further functional analysis showed over-expression in both the w-GR and w-CAL populations (Figure 4H).

#### Chitinase 5 (Cht5)

In insects, chitin is known as a scaffold material, providing both exo- and endo-support to the cuticles of the epidermis and trachea as well as the peritrophic matrices lining the gut epithelium [97]. The midgut *chitinases* seem to be involved in the formation, perforation and degradation of the midgut peritrophic matrix, which protects the gut epithelium from damaging factors, toxins and pathogens [98–100]. *Chitinases* have also been proposed as biopesticides, as transgenic plants expressing chitinolytic enzymes potentiate the efficacy of other biological toxins (e.g. Bt or fungal toxins) [101, 102]. In the olive fly, *Cht5* was under-expressed in the SPIN transcriptome and was found down-regulated in the w-CAL populations (Figures 2 and 4M). Given the aforementioned role of *chitinases*, we can hypothesize that by under-expressing *chitinase* genes the resistant flies decrease spinosad penetrance, thus increasing resistance.

#### Cell division cycle-associated protein 7 (Cdc)

This gene belongs to the HAD-superfamily hydrolase, according to Interpro [103]. RNAseq analysis showed that *Cdc* was under-expressed in the SPIN transcriptome. However, after functional analysis the RNAseq result was not confirmed, since both the resistant w-CAL population and the sensitive w-GR were up-regulated compared to the sensitive LAB flies (Figure 4P). Therefore, further analysis is required in order to clarify *Cdc’s* role in spinosad resistance.

### RNA viral genes

Five more genetic loci were of curious origin. Two of them were up-regulated: a *replicase-like protein* was identified as having considerable similarity with a dimethyl transferase domain of an RNA virus; and an elongation factor had similarity with a viral helicase domain. Three of them were down-regulated (*hypothetical B. oleae polyprotein; RNA-dependent RNA polymerase*; *microtubule-actin cross-linking factor 1*), but they are also implicated with viral functions as homology searches matched sacbrood virus sequences. Finding similarities with viral sequences is not surprising. In fact, the presence of viral sequences has been reported in previous both smaller and larger transcriptome sequencing efforts [41, 104, 105]. Obviously, such genes reflect the presence of RNA virus infections in different laboratory or wild populations. The impact of such infections has not been studied and cannot be assessed at this point whether this might have been among the causes of our SPIN colony collapse.

## Conclusion

Adaptation and survival of the flies in the altered environment caused by insecticide stress appears to be a consequence of changes in multiple genes’ expression, affecting both biological and physiological pathways. Our perception about the development of insecticide resistance in insects, traditionally attributed to either a target site alteration or the up-regulation of various detoxification genes (such as *P450s, esterases* and *GSTs*), is recently changing due to our ability to address such questions in a more holistic way through transcriptomic analysis. This gives us the opportunity to consider diverse regulatory networks of interacting genes via complex mechanisms. In the present study, we conducted whole transcriptome comparative analyses between spinosad resistant and susceptible olive flies, in order to investigate and identify genetic loci and molecular mechanisms that are most likely to be involved in spinosad resistance. The observed changes at the RNA level as well as the functional analyses and bioassays, point towards a multi-level impact of the insecticide to the insect’s physiology. Our results indicate that the organism’s response to this novel environmental stressor mainly affects energy metabolism pathways, immunity defense pathways and detoxification. The oxidative, xenobiotic, and innate immune stress response pathways appear to be coordinated, leading to the regulation of numerous cellular and biological/physiological processes. Further studies are required to determine the molecular mechanisms and significance of this cross-regulation.

## Methods

### Ethics statement

The study was carried out on laboratory reared olive flies and wild olive flies collected from the area around the city of Volos, Greece, and the Sonoma County in California. No specific permissions are required for these experiments or collections, since these studies did not involve endangered or protected species.

### Fly culture and stocks

#### Laboratory strain

The laboratory strain of the olive fly (LAB) is part from the original stock from the Department of Biology, ‘Demokritos’ Nuclear Research Centre, Athens, Greece, and has been reared in our laboratory for over 15 years. The flies are reared at 25°C with a 12 h light/12 h dark photoperiod in 30 × 30 × 30cm^3^ cages, as described by [106–108].

#### Development of a spinosad-resistance colony

A spinosad resistant strain (SPIN) was also developed in our laboratory. Starting material for this colony was the aforementioned LAB colony that was supplemented with ~1000 wild flies from Argalasti (Pelion, Greece). Increasing amounts of spinosad were gradually introduced into the colony’s feeding water that reached 0.04 g/ml after 10 generations. The colony was maintained for about 22 generations (~2 years) under constant 0.04 g/ml spinosad selection. This amount of spinosad corresponds to approximately 2× the recommended amount for field applications that would result in 100% mortality. It also corresponds to 125× the LC50 of the susceptible LAB strain. In order to increase the resistance to spinosad, the colony was refreshed a second time with wild flies from Sonoma County (CA, USA), since this area was shown to have the highest spinosad resistance level [109]. Six months later the colony practically crashed and was recovered by a single female, under no selection. Progeny of that female were put under gradually increasing amounts of spinosad. The colony recovered previous levels of resistance (0.04 g/ml) after only 4 generations. After a total of 46 generations, a more precise estimation of the resistance ratio (RR) was obtained by ingestion bioassays, as described in Kakani et al. [109], showing that resistance level had reached 35×. This is the stage from where all spinosad resistant laboratory flies (referred to as SPIN throughout the text) were collected, both for the isolation of the nAChR and the RNAseq analysis. Finally, during the fall of 2012, entirely unexpectedly and without any obvious changes in the insectary environment, the spinosad resistant colony crashed. Initially it was noted that females did not oviposit in the offered waxed cone, while both male and female adult numbers started to decline. During that time, new wild material arrived from California, which was intended to enrich the laboratory colony with new alleles. Nonetheless, after about 3 months of continuous efforts the last adult flies died and no progeny emerged.

#### Field-collected flies

Wild flies were collected from two geographical locations, one from an untreated orchard in Greece [Agria, Pelion (w-GR)] and another from a different site in Sonoma County [CA, USA (w-CAL)] that was the source of flies used to refresh the SPIN strain, but where flies had also shown highest levels of spinosad resistance in the Kakani et al. study [109]. Contact bioassays were performed on these flies according to Kakani et al. [109], using seven doses of spinosad ranging between 1/2× to 1/128×, plus a blank control of acetone. LD_50_ values and 95% confidence intervals were calculated by probit analysis using SPSS v.13 (SPSS Inc, Chicago, IL). The calculated resistance ratio (RR) of the w-CAL was 12.96 (11.62-14-28) whereas that of the w-GR was 3.14 (2.25-4.2). Infected olives were brought into the laboratory and emerged flies were put in 30 × 30 × 30cm^3^ cages and fed on the standard yeast hydrolysate diet [107]. Female flies were allowed to oviposit in fresh olives, since wild olive flies do not oviposit on artificial substrates. Flies from this F1 generation were used for the functional analysis experiments described in the Results.

### Extraction of RNA, cDNA synthesis, cloning of nAchR Boα6 and sequencing

Total RNA was isolated from pools of four heads of adult flies from the LAB and SPIN strains with the use of TRIzol® Reagent (Ambion-Invitrogen). One to five micrograms of total RNA was used for first strand synthesis of poly(A) of cDNA using the MMLV high performance Reverse Transcriptase (GeneOn) and random primers (GeneOn) according to the manufacturer’s instructions.

Partial cDNA of the LAB acetylcholine nicotinic receptor α6 gene of *B. oleae* was amplified by PCR using primers Bdα6-F (ACATGGTTCCCATTCGATGACC) and Bdα6-R (GCGACCATGAACATGATGCAATT) designed on conserved regions of the published nAChRα6 cDNA sequence of *Bactrocera dorsalis* (Bdα6-JN560169.1) [26]. The PCR amplification reaction consisted of 2 μl of the first strand cDNA reaction mix as a template, 0.7 μl of 10 mM primers, 0.2 mM dNTPs, 1.5 mM MgCl_2_ and 1unit Taq DNA Polymerase (GeneOn) in a 20 μl reaction. Cycling conditions were 95°C for 5 min, followed by 30 cycles of 95°C for 30s, 49°C for 2 min and 72°C for 1.5 min and a final extension at 72°C for 10 min in a thermal cycler (MJ Mini Biorad). The amplified PCR product was then separated in a 1% agarose gel, stained with ethidium bromide. The amplified PCR product was isolated by the GF-1 Gel recovery kit (Vivantis) and subcloned into the pBluescriptII SK(+) plasmid vector and sequenced. Based on the obtained sequence, four gene specific primers were designed to amplify the full-length cDNA: two reverse primers for 5′-RACE PCR (5GSP1: 5′- GTCCTTAGATTTCAGCTACC-3′ for the first round reaction and 5GSP2: 5′-GGGCGGGTGGGTATAAGTAT-3′ for the nested reaction) and two forward primers for 3′-RACE PCR (3GSP1: 5′- CACAACGGTGGAGGAGCATC-3′ for the first round reaction and 3GSP2: 5′-GGGCGGGTGGGTATAAGTAT-3′ for the nested reaction). A poly-A tail was added to the 3′-end of the resulting strand of 5′-RACE by terminal deoxynucleotidyl transferase (TdT, Biolabs). Thermal cycling conditions for the 5′- and 3′-RACE were: pre-denaturation 5 min at 94°C, 30 cycles of 94°C for 30 sec, 49/52°C (first/second round) for 45 sec and 72°C for 2 min (according to the size of the expected fragment) with a final extension of 15 min at 72°C. The resulting PCR products of 5′-RACE and 3′-RACE were subcloned into pBluescriptII SK (+) vector and sequenced. Each time plasmids were sequenced, three different isolates were used and no variation was observed.

### Sequence comparison between sensitive and resistant *Bactrocera oleae*nAChRα6 subunits

For comparison of the *Boα6* transcripts, total RNA was extracted from a pool of 4 adult heads from the two strains (LAB and SPIN), as described above. The specific primer pair Boα6-F (5′-AGATTAGTGACAGCATAACCG-3′) and Boα6-R (5′-TCTATCCACAACCATTGCCGC-3′) was used for the amplification of the full-length open reading frame of BoAChR-α6 gene. The PCR products were sequenced directly with the use of Boα6-F, Boα6-R and two more internal primers (Boα6F1: 5′-ATGAATCGGAATATGGAG-3′ and Boα6R1: 5′-AACGGATTTAATCCAAGG-3′). No multiple peaks were observed in the obtained sequences, indicating the absence of sequence polymorphism in the pools.

Nucleotide sequence similarity searches were performed using BLAST [110]. Multiple sequence alignments [111] with other insect nAChR subunits were performed with ClustalW2 [112]. The calculated molecular weight and isoelectric point of the putative protein encoded by *Boα6* were predicated by Compute pI/Mw tool in Expasy Server [113]. Phosphorylation sites and N-linked glycosylation sites were identified by the PROSITE database [114].

### RNA isolation for library preparation and functional analysis

Total RNA was isolated from fly heads with the use of TRIzol® Reagent (Ambion-Invitrogen) following the instructions of the manufacturer with minor modifications. More specifically, RNA was extracted from forty male and forty female heads from the laboratory colony (LAB) and from an equal number of spinosad resistant fly heads (SPIN). For more complete sequence assembly, two more libraries were constructed and sequenced: a FEMALE library made of female accessory glands and spermathecae of ~300 female flies and a MALE library made of testes of ~150 male flies [115]. RNA extraction was followed by an additional DNA removal using the TURBO DNA-free Kit (Ambion-Invitrogen), according to manufacturer’s instructions. The integrity of RNA was assessed by 1% agarose gel electrophoresis and the purity of all RNA samples was evaluated at Fleming Institute (Greece) with the use of (Agilent 2100 Bioanalyzer) and NanoDrop (2000).

For functional analysis, RNA was extracted as described above from three different individual male and female heads from the LAB strain, the SPIN resistant strain, the Sonoma County wild population (w-CAL) and the Agria (w-GR) wild population.

### Whole transcriptome library preparation for next-generation sequencing with the SOLiD 4 Sequencing System

RNA transcripts expressed in the head of the spinosad-sensitive (LAB) and spinosad-resistant (SPIN) olive fly strains were used to construct cDNA library for high throughput sequencing analysis on the SOLiD 4 Sequencing System. More specifically, polyadenylated RNA (polyA-RNA) was isolated from 5 μg of total RNA using the Dynabeads Oligo(dT) kit (Ambion, Life Technologies Corporation). The isolated polyA-RNA was randomly fragmented by chemical hydrolysis at 94°C for 5 minutes and was then treated with antarctic phosphatase to remove phosphate groups from the fragments’ ends, followed by treatment with T4 polynucleotide kinase to add a Pi at the 5′ end of each fragment. The resulting RNA fragments were hybridized and ligated to the P1 and P2 adaptor sequences specifically designed for sequencing with the SOLiD system (SOLiD Total RNA-Seq Kit, Life Technologies Corporation). The RNA produced was reverse transcribed to cDNA which was then amplified in a 15-cycle PCR. At this step, the use of different barcoded 3′ PCR primers from the selection included in the SOLiD barcoding kit allowed the preparation of cDNA libraries for multiplex sequencing. From the cDNA produced, only fragments of average size 200–300 bp were selected with two rounds of magnetic bead purification (Agencourt AMPure XP Reagent, Beckman Coulter).

The quality and size of the purified cDNA library was assessed on the Agilent Bioanalyzer 2100 (Agilent Technologies Inc.) and with quantitative PCR using the Library Quant Kit ABI Solid (KAPA Biosystems). A multiplex library mix (500pM) was used to prepare a full-slide for analysis on the SOliD 4 Sequencing System (Applied Biosystems) with 35 + 50 bp PE –chemistry.

### Bioinformatics analysis

The reads of the libraries were assembled to construct the reference transcriptome using the SOAPdenovo assembler [39] with a word size of 25 nt and using all paired and unpaired reads. Annotation of the assembled sequences was obtained by aligning against the NCBI non-redundant (Nr) protein database using blastx [116] and collecting the annotations with the BLAST2GO tool [40]. TopHat [117] was used to generate a spliced alignment to the reference transcriptome. Transcripts were assembled using Cufflinks and Cuffdiff [43] was used in order to reveal differentially expressed genes. SNP calling was performed with the mpileup tool and converted to the vcf fomat using the vcfutils, both from the SAMTOOLS package [42]. The SNP loci were intersected with the gene coordinates using the intersectBed tools from the BEDtools suite [118].

### Expression stability of candidate reference genes in *B. oleae*head

In order to find the most suitable reference gene for gene expression analyses in *B. oleae* head tissue, nine different housekeeping genes commonly used in other dipteran species were analyzed. The nine genes were: *RPL19* (*ribosome protein L19*), *tbp* (*TATA-binding protein*), *ubx* (*ultrabithorax*), *GAPDH* (*glyceraldehyde 3-phosphate dehydrogenease*), *α-TUB* (*α-tubulin*), *β-TUB* (*β-tubulin*), *14-3-3zeta*, *RPE* (*RNA polymerase II*) and *actin3.* To determine the expression stability of the selected genes in *B. oleae* head, the expression of the reference genes was measured in 24 heads (6 individuals from each of the LAB, SPIN, w-GR and w-CAL populations, i.e., 24 biological replicates) in duplicate reactions (two technical replicates). The amplification efficiency of the reactions was calculated by the CFX Manager™ software (Bio-Rad) (Additional file 1: Table S4). Using the comparative Cq method with a procedure of specific PCR efficiency correction, all the Cq values were converted to relative quantities and transformed to an input file format with raw data for subsequent analysis by the Normfinder Excel applications.

Normfinder [119] is an algorithm for identifying the optimal normalization gene among a set of candidate genes. This software is based on a mathematical model of gene expression that enables estimation not only of the overall variation of the candidate normalization genes but also of the variation between samples subgroups of the sample set [44].

BestKeeper determines the most stably expressed genes based on the coefficient of correlation to the BestKeeper Index, which is the geometric mean of the candidate reference gene Cq values. Additionally, it calculates the standard deviation (SD) and the coefficient of variation (CV) based on the Cq values of all candidate reference genes [45]. Reference genes are identified as the most stable genes, i.e. those that exhibit the lowest coefficient of variance and standard deviation [120].

Additional file 1: Table S5 presents the data on the ranking of the tested reference genes.

### Functional analysis of spinosad-resistance differentially expressed genes

Specific primers for the amplification of the differentially expressed genes revealed by the transcriptome analysis were designed by Primer-BLAST [121] (Additional file 1: Table S4).

For the functional analysis experiments, RNA was extracted from the heads of six individual flies (equal number of males and females) of all different strains and populations described previously. Subsequently, one microgram of each DNA-free total RNA was converted into cDNA using 300 ng Random hexamer primers (equimolar mix of N_5_A, N_5_G, N_5_C and N_5_T), 200 units MMLV Reverse Transcriptase (Geneon), 5X reaction buffer, 40 mM dNTP mix and 40 units RNase Inhibitor (GeneOn) according to the manufacturer’s instructions.

Relative quantitation was used to analyze changes in expression levels of the selected genes using a Real-time PCR approach. Expression values were calculated as the geometric mean of the relative expression of each target gene against the expression of each one of the reference genes *tbp* and *14-3-3 zeta* gene. The qRT-PCR conditions were: polymerase activation and DNA denaturation step at 95°C for 4 min, followed by 40 cycles of denaturation at 95°C for 30 s, annealing/extension and plate read at 56°C for 30 s and finally, a step of melting curve analysis at a gradual increase of temperature over the range 55°C to 95°C. In this step, the detection of one gene specific peak and the absence of primer dimer peaks was assured. Each reaction was performed in a total volume of 15 μl, containing 5 μl from a dilution 1:10 of the cDNA template, 1X iTaq Universal SYBR Green Supermix (Bio-Rad) and 400 nM of each primer. The reactions were carried out on Bio-Rad Real-Time thermal cycler CFX96 (Bio-Rad, Hercules, CA, USA) and data analysed using the CFX Manager™ software. All assays were performed three times (three technical replicates), contained six different individuals (six biological replicates) and three negative controls. A standard curve was generated for each gene using 5-fold serial dilutions of pooled cDNA from the flies head. The PCR efficiency (E) and the correlation coefficient (R^2^) characterizing each standard curve are given in Additional file 1: Table S4. Efficiencies for all tested genes varied between 93.3% to 109.2%. The 2^-ΔΔCt^ method was used for the analysis of relative gene expression [122].

### String analysis

In order to investigate the potential interactions between the up- and down-regulated genes, we queried the resource STRING (Search Tool for the Retrieval of Interacting Genes) which makes available precomputed results in predicted functional linkages among proteins by comparative genomics and text-mining [46]. Specifically, the gene IDs of the *Drosophila melanogaster* orthologs of our genes were used as input in the online database resource STRING in order to be placed in a biological context according to a large number of computational predicted and experimentally determined functional associations and protein-protein interactions. Results were graphically displayed and scored using a STRING specific scoring scheme that correlates with validated protein-protein functional associations.

### Statistical analysis

Statistical analysis was performed using GraphPad Prism 6 [123] after normalization of raw Cq values. The normality for all genes was based on the Kolmogorov-Smirnov and Dallal-Wilkinson-Lillie tests (alpha = 0.05). For the genes that passed the normality test, one-way ANOVA and the Tukey’s multiple comparison tests were performed. Genes that did not pass the normality test were analyzed by the non-parametric Kruskal-Wallis test with P < 0.05.

### Availability of supporting data

All data have been deposited at the Sequence Read Archive (SRA) of NCBI. All reads for each sample are summarized at the BioProject page: http://www.ncbi.nlm.nih.gov/bioproject/PRJNA231981.

## Electronic supplementary material

Additional file 1: Table S1: Polymorphic sites in the nAChR α6-subunit sequences in the olive fly LAB and SPIN strains **Table S2.** Up- and down-regulated genes in spinosad resistant olive fly heads. **Table S3.** Basic statistics of relative expression of the up- and down-regulated genes. **Table S4.** Primer sequences used for q-PCR. **Table S5.** Normfinder and Bestkeeper analysis results. (XLSX 21 KB)
